# Mathematical modeling of Notch dynamics in *Drosophila* neural development

**DOI:** 10.1080/19336934.2021.1953363

**Published:** 2021-10-05

**Authors:** Tetsuo Yasugi, Makoto Sato

**Affiliations:** a Mathematical Neuroscience Unit, Institute for Frontier Science Initiative, Kanazawa University, Kanazawa, Ishikawa, Japan; b Laboratory of Developmental Neurobiology, Graduate School of Medical Sciences, Kanazawa University, Kanazawa, Ishikawa, Japan

**Keywords:** Notch signalling, lateral inhibition, mathematical modelling, neuroblasts, sensory organ precursors, eye development, proneural wave

## Abstract

Notch signalling is a well-conserved signalling pathway that regulates cell fate through cell-cell communication. A typical feature of Notch signalling is ‘lateral inhibition’, whereby two neighbouring cells of equivalent state of differentiation acquire different cell fates. Recently, mathematical and computational approaches have addressed the Notch dynamics in *Drosophila* neural development. Typical examples of lateral inhibition are observed in the specification of neural stem cells in the embryo and sensory organ precursors in the thorax. In eye disc development, Notch signalling cooperates with other signalling pathways to define the evenly spaced positioning of the photoreceptor cells. The interplay between Notch and epidermal growth factor receptor signalling regulates the timing of neural stem cell differentiation in the optic lobe. In this review, we summarize the theoretical studies that have been conducted to elucidate the Notch dynamics in these systems and discuss the advantages of combining mathematical models with biological experiments.

## Introduction

Notch signalling is conserved across metazoans and plays a central role in cell fate decisions [1,2]. Notch was genetically identified in *Drosophila* with the ‘notching’ mutant phenotype in the wing more than 100 years ago [3]. Subsequent analyses in *Drosophila* have uncovered the Notch receptor and the other key molecules that constitute the Notch signalling pathway. One of the key processes in Notch signalling is the lateral inhibition system. Typical examples of lateral inhibition have been described in *Drosophila* neural stem cell (neuroblast, NB) differentiation during embryogenesis and sensory organ precursor (SOP) specification in the thorax [4–6]. The initially uniform field of cells acquires either neuronal or non-neuronal fate through lateral inhibition. In addition to the conserved idea of lateral inhibition, many studies using *Drosophila* and other animals have revealed diverse functions of Notch signalling in development, tissue homoeostasis, and cancer [7,8].

**Figure 1.**
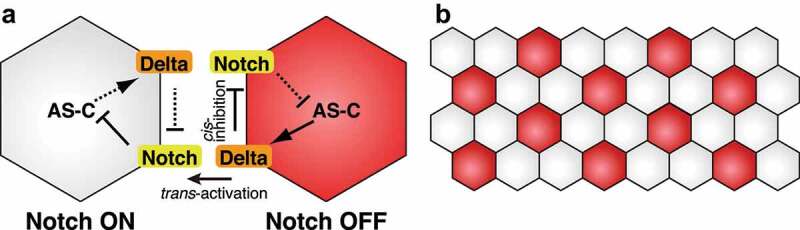
Mathematical modelling of neuroblast and sensory organ precursor differentiation. (a) Notch-mediated lateral inhibition. Delta expression in the right cell activates Notch signalling in the left cell, which leads to downregulation of *AS-C* expression. Since AS-C induces *Delta* expression, Delta expression gradually decreases in the left cell. In contrast, Delta expression inhibits Notch signalling in the same cell. This leads to the activation of *AS-C* and *Delta* expression in the right cell. (b) The salt-and-pepper-like pattern generated by the Notch function. Neural cells (red) such as embryonic NBs and SOPs are not next to each other

In this review, we will not mainly focus on the molecular mechanisms of Notch signalling because it has been well documented in several review articles. [9–14]. Here, we simply abstracted the essential factors required for the canonical lateral inhibition system in *Drosophila* (Figure 1a). Notch is a single-pass transmembrane receptor [15]. There are two membrane-bound Notch ligands in *Drosophila*: Delta and Serrate [16–18]. Binding of the Notch ligand expressed in neighbouring cells to Notch leads to a conformational change in Notch, and the intracellular domain of Notch (NICD) is cleaved by γ secretase [19]. NICD translocates to the nucleus and activates target gene expression together with Suppressor of Hairless (Su(H)) and Mastermind (Mam) [20–23]. The NICD-Su(H)-Mam complex induces the expression of *enhancer of split* (*E(spl)*) complex genes, which encode basic helix-loop-helix (bHLH) transcription factors [20,24,25]. E(spl) transcription factors inhibit the expression of *achaete-scute* complex (*AS-C*), which includes *achaete* (*ac), scute* (*sc), lethal of scute* (*l’sc*), and *asense* (*ase*) [26,27]. AS-C genes encode bHLH transcription factors and act as proneural factors. Additionally, AS-C induces Delta expression [28]. The neighbouring cells differentiate into different cell types based on this feedback mechanism. This effect is called as *trans*-activation. In contrast to *trans*-activation, *cis*-inhibition negatively regulates Notch signalling. In *cis*-inhibition, Notch ligands and the Notch receptor expressed in the same cell bind with each other and inactivate Notch signalling [29–32]. In the wing disc development, high levels of Delta and Serrate inhibit activation of Notch signalling in a cell-autonomous manner through *cis*-inhibition [32,33]. Although it is unclear whether *cis*-inhibition is implemented in all biological systems involving Notch signalling, *cis*-inhibition enhances the effect of *trans*-activation and contributes to generating mutually exclusive cell states between neighbouring cells during lateral inhibition as explained below.

Recently, formulation of mathematical models of Notch signalling to understand its dynamics has become popular [34]. In this review, we focus on the developmental processes of *Drosophila*, in which Notch signalling plays pivotal roles through lateral inhibition. The basic concept of lateral inhibition was identified by investigating the mechanism of NB formation during embryonic stages and SOP formation in the thorax [4–6].

However, in many other biological systems, Notch signalling cooperates with other signalling pathways and shows complicated interactions. Such examples are observed in eye disc and optic lobe development. In eye disc development, sequential propagation of the morphogenetic furrow induces photoreceptor cell (R cell) differentiation [35,36]. Differentiation of R cells resembles that of embryonic NBs and SOPs in that only a small number of cells are selected as photoreceptor neurons from initially equivalent epithelial cells. Notch signalling, together with the Hedgehog (Hh), Decapentaplegic (Dpp), and epidermal growth factor receptor (EGFR) pathways, defines the evenly spaced positioning of the R cells.

During optic lobe development, a wave of differentiation named ‘proneural wave’ sweeps the surface of the neuroepithelium and determines the timing of NB differentiation [35,37]. In the optic lobe, all neuroepithelial cells (NEs) sequentially differentiate into NBs following the proneural wave. The interaction between Notch-mediated lateral inhibition and EGFR-mediated reaction diffusion determines the speed of proneural wave progression. In these systems, it is difficult to explain Notch dynamics without theoretical approaches because these signalling pathways show complicated interactions. In this article, we introduce molecular bases of these developmental processes and mathematical approaches to decipher diverse Notch functions.

## Mathematical models of canonical Notch-mediated lateral inhibition

The basic idea of Notch-dependent lateral inhibition comes from studies of NB differentiation in the embryonic neuroectoderm and SOP selection in the thorax [4–6]. In these biological systems, proneural genes of the *AS-C*, including *ac, sc*, and *l’sc*, are expressed in proneural equivalence groups of neuroectodermal cells, called proneural cluster cells, and have the potential to differentiate into neural cells [38–40]. Among proneural cluster cells, single cells are selected as neural cells, and other cells are fated to be epidermal cells [41,42]. This bistable specification is explained by the Notch-mediated lateral inhibition [9,43]. During the specification step, binding of Delta to the Notch receptor in the neighbouring cell activates Notch signalling and inhibits *AS-C* and *Delta* expression (Figure 1a). As a result, these cells do not differentiate into neural cells. In contrast, Delta-expressing cells maintain the expression of AS-C proneural factors to be neural cells. The feedback loop amplifies the slight initial differences in Delta and/or AS-C expression and dictates different cell fates between adjacent cells (Figure 1a). According to the lateral inhibition mechanism, neural cells are not adjacent to each other and show a so-called salt-and-pepper-like pattern (Figure 1b).

To understand the function of lateral inhibition during the specification of embryonic NBs and thoracic SOPs, mathematical and computational approaches have been taken [44–50]. A simple model only considers the differentiation state of the cells; differentiation states of cells were calculated according to the lateral inhibition rule under the assumption that if a cell has acquired a neural fate, then the differentiating cell inhibits the neural differentiation of neighbouring cells. This simple model reproduced the differentiation pattern and the ratio of neural and non-neural cells [44,45]. Collier *et al*. provided a two-component model to explain the function of Notch signalling in the lateral inhibition mechanism [48]. The model considered the levels of Notch activation (*N*) and Delta expression (*D*). *N* is increased by the effect of *trans*-activation through *D* expressed in neighbouring cells and decreased by its degradation. *D* is decreased by *N* in the same cell and is regulated by decay. Numerical simulations of the model demonstrated a spatially periodic pattern in which cells with low *N* are not adjacent to each other and are surrounded by cells with high *N*, reproducing the salt-and-pepper-like differentiation pattern *in vivo*. Additionally, mathematical models that include the effect of *cis*-inhibition efficiently amplified small initial differences between neighbouring cells and accelerated the pattern formation by lateral inhibition [31,51]. These mathematical models and numerical simulations clearly show that Notch-dependent lateral inhibition can determine different cell fates between neighbouring cells.

Recent studies combining mathematical approaches and *in vivo* analyses have revealed that Notch signalling mediates the tissue-wide patterning of SOPs in the notum. It has been thought that SOP selection includes two distinct steps. First, the expression of proneural factors is regulated by developmental signals that convey positional and temporal information. In the next step, Notch-mediated lateral inhibition singles out SOPs among the proneural cluster cells. In contrast to the two-step model, Notch signalling governs both proneural patterning and SOP selection in the medial thorax [52]. The initial expression pattern of Delta and subsequent self-organized Notch dynamics organizes stereotyped SOP patterning. In addition, Cohen *et al*. showed that basal actin-based filopodia can transmit intermittent Notch signalling over several cell diameters to adjust the number and spacing of SOPs [53]. These findings suggest that the dynamic action of Notch signalling regulates tissue-wide patterning.

## Notch function in eye disc development

**Figure 2.**
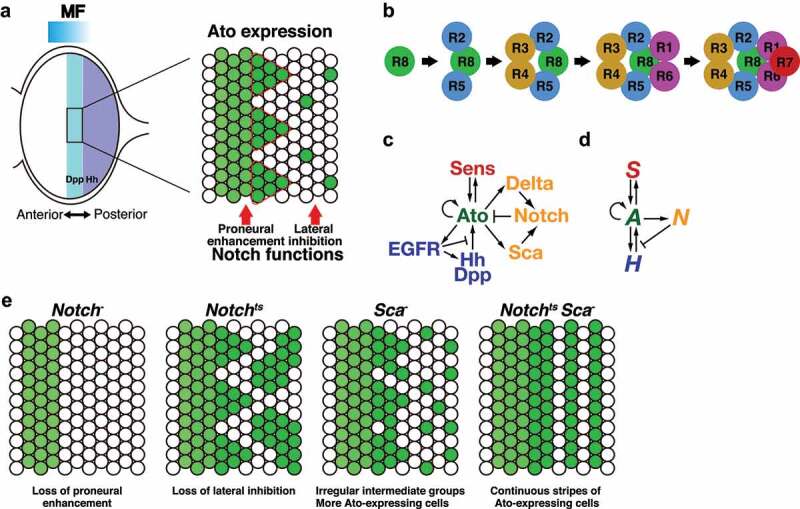
Mathematical modelling of the eye disc development. (a) Development of the eye disc. The morphogenetic furrow (MF) progresses in the posterior to the anterior direction (left). Hh is expressed in the differentiating R cells located posterior to the MF. Dpp expression shows a stripe in the MF. Atonal (Ato, green) shows dynamic expression patterns during the eye disc development (right). Notch signalling involves early ‘proneural enhancement’ and late ‘lateral inhibition’ functions. Cells of intermediate groups are circled in red. (b) Recruitment of R cells in the ommatidium. (c) Genetic interaction that regulates Ato expression (modified from Lubensky *et al*., 2011) [85]. (d) A simplified interaction map of (B) for mathematical modelling of Ato expression (modified from Lubensky *et al*., 2011) [85]. *A, S, H*, and *N* represent activation signals in the cell, delayed positive feedbacks in the cell, long-range cell-non-autonomous activation signals, and cell-non-autonomous inhibitory signals, respectively. (e) Schematics of the Ato expression pattern in *Notch^−^, Notch^ts^, Sca^−^*, and *Notch^ts^ Sca^−^* mutant conditions

The adult eye in *Drosophila* consists of 750 spatially arranged ommatidia. Each ommatidium comprises eight R cells (R1-8) and several types of accessory cells. Differentiation of R cells starts from the posterior margins of the eye disc and progresses towards the anterior side [35,36,54]. The morphogenetic furrow (MF) is the site where R cell differentiation is initiated (Figure 2a) [55]. The posterior to anterior progression of the MF is driven by the secreted Hh protein expressed in the differentiated R cells (Figure 2a) [56–58]. In each ommatidium, differentiation of R8 occurs first, followed by R2/R5, R3/R4, and R1/R6 differentiation (Figure 2b). R7 is recruited at the end (Figure 2b). Therefore, patterned differentiation of R8 cells is important for generating a crystalline hexagonal array of the adult eye. Instead of AS-C proneural factors, another bHLH proneural factor, Atonal (Ato), plays a central role in R8 differentiation [59–61]. Ato is expressed in a broad stripe within and just anterior to the MF (Figure 2a). As the MF passes anteriorly, Ato expression is elevated in the regularly spaced intermediate groups. Later, Ato expression is refined to R8 only and shows a more evenly spaced pattern (Figure 2a). Several factors and signalling pathways regulate Ato expression and R8 specification. Dpp signalling, together with Hh signalling, induces Ato expression at the MF (Figure 2a, c) [62–65]. The Notch function in Ato expression is complex [66]. Loss-of-function of *Notch* does not show elevation of Ato expression, suggesting that Notch signalling is required for establishing a high level of Ato expression in the early step (Figure 2e) [66–69]. This function of Notch signalling is called ‘proneural enhancement’ (Figure 2a). Conversely, in the next step, Notch signalling restricts the number of Ato-expressing cells by lateral inhibition (Figure 2a, c) [66,70,71]. The number of Ato-expressing cells is increased when Notch signalling is partially perturbed by using *Notch^ts^*, a temperature-sensitive mutation of *Notch* (Figure 2e) [72]. A secreted glycoprotein, Scabrous (Sca), regulates R8 specification by activating Notch signalling (Figure 2c) [73–76]. Ato induces Sca expression, and the secreted Sca binds to the Notch receptor to inhibit Ato expression [71,72,77,78]. In *Sca* mutant eye discs, intermediate groups are not formed correctly and more R8s are generated (Figure 2e) [71,75]. Since Sca acts as a diffusible protein, it is expected that Sca mediates Notch-mediated lateral inhibition in several cell diameters for regular patterning. In addition to Notch signalling, EGFR signalling also participates in R8 spacing [79,80]. EGFR signalling also activates Hh signalling (Figure 2c) [81,82]. Ato induces the expression of a transcription factor, Senseless (Sens), to induce R8 differentiation [83]. Sens, in turn, maintains Ato expression in presumptive R8 cells (Figure 2c).

Based on the above biological evidence, Pennington and Lubensky developed a mathematical model to understand the mechanism of MF progression and spatial R8 patterning [84]. The authors abstracted molecular interactions controlling Ato expression and set three variables: cell-autonomous activators (*A*), cell-non-autonomous activators (*H*), and cell-non-autonomous inhibitors (*N*). *A* includes the functions of Ato and Sens. *H* represents the functions of Hh and Dpp signalling, which drives the MF anteriorly and induces Ato expression. All cell-non-autonomous inhibitory signals, including Notch signalling and Sca functions, were integrated into the variable *N*. Numerical simulation of the simplified model recapitulated the MF propagation and R8 specification [84]. This result suggests that these interactions among the three components are sufficient for stable and stationary patterning in the eye disc. Lubesnsky *et al*. revised this model by separating the functions of Ato and Sens (Figure 2c, d) [85]; *A*, and *S* represent the function of Ato and Sens, respectively. The revised model also uses the variables *H* and *N*, as described in the original model. Simulations based on the four-component model produced continuous stripes of R8s when the effect of lateral inhibition by *N* was slower than that in wild type situations. Such striped patterns were experimentally reproduced by combining *Notch^ts^* and *Sca* mutations (Figure 2e) [85]. Zhu *et al*. established a computational model by further describing the activity of the Hh, Dpp, Notch, and EGFR pathways in R8 specification [86]. Simulations of the computational model showed the robust spatio-temporal order in R8 patterning and revealed that the coupling between long-range inductive signals by Hh and the short-range restrictive signals by Notch and EGFR is important for the accurate spacing of R8s. To elucidate the precise Notch dynamics in eye disc development, the early function of Notch in proneural enhancement and its late function in lateral inhibition need to be separately examined. Future mathematical models that separate these opposing functions of Notch signalling together with *in vivo* experiments will provide a better understanding of spatio-temporal Notch dynamics in eye disc development.

## Notch function in optic lobe development

**Figure 3.**
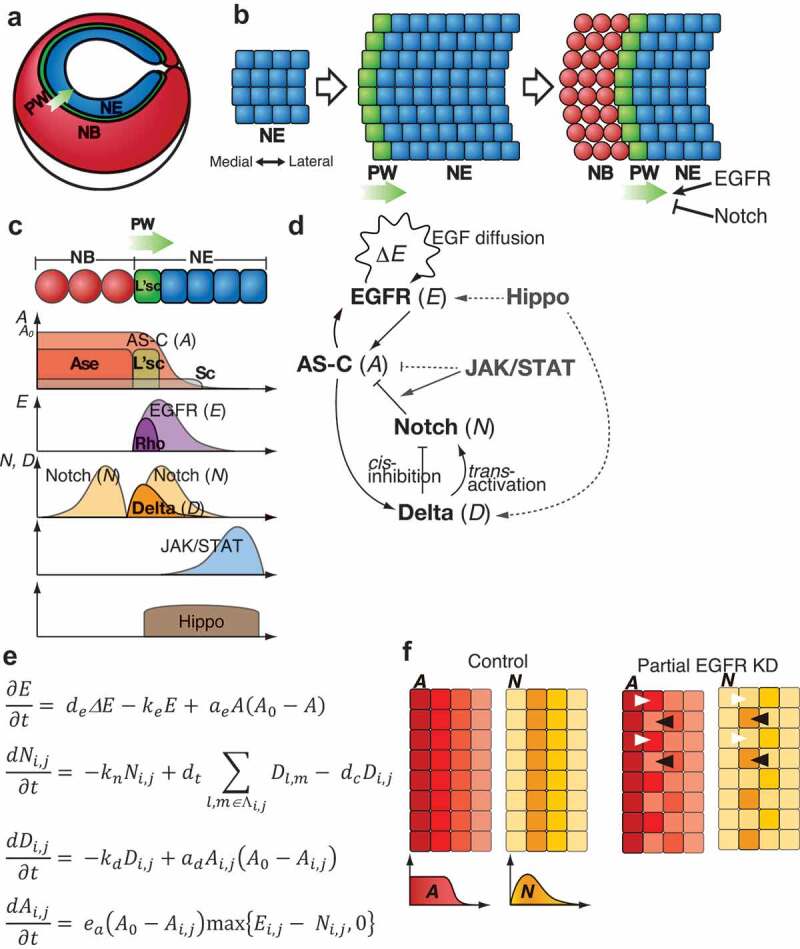
Mathematical modelling of the proneural wave progression. (a) Schematic representation of the optic lobe. Neuroepithelial cells (NE), neuroblasts (NB), and the proneural wave (PW) are shown. (b) Development of the optic lobe. The proneural wave progresses in the medial to the lateral direction. EGFR and Notch signalling positively and negatively regulate the wave progression, respectively. (c) Activation of AS-C, EGFR signalling, Notch signalling, JAK/STAT signalling, and Hippo signalling during the proneural wave progression. (d) Genetic interaction among AS-C, EGFR signalling, and Notch signalling, JAK/STAT signalling, and Hippo signalling. (e) The mathematical model of the proneural wave including the interaction among AS-C, EGFR signalling, and Notch signalling (modified from Sato *et al*., 2016) [111]. (f) Schematics of the simulation results of control (left) and partial EGFR knockdown (right). Activation of *A* and *N* is shown. White and black arrowheads represent cells with high *A* and high *N* levels, respectively

Another unique function of Notch dynamics is found in the developing optic lobe [35,37,87–92]. The adult optic lobe is composed of four ganglia: the lamina, medulla, lobula, and lobula plate. Among these, the medulla is the largest component, and most of the medulla neurons are generated from NEs in the outer proliferation centre (OPC) during the larval and early pupal stages. During the early stages of larval development, NEs in the OPC proliferate by symmetric cell division (Figure 3a, b) [93]. Later, differentiation from NEs to NBs starts from the medial edge of the optic lobe and progresses in the medial to lateral direction (Figure 3a, b) [94,95]. Proneural factors including Sc, L’sc, and Ase are expressed in wavefront cells and differentiated NBs, and determine the timing of differentiation (Figure 3c) [94–96]. Therefore, the wave of differentiation is named as the ‘proneural wave’ [95]. Among the proneural factors, L’sc is transiently expressed in wavefront cells and thus used as a marker for the proneural wave [95]. It has been reported that several conserved signalling pathways, such as Notch, EGFR, JAK/STAT, and Hippo regulate the proneural wave progression. Delta is temporally expressed in the wavefront cells (Figure 3c). Notch signalling shows two activation peaks, once in wavefront cells and again in NBs (Figure 3c). The first Notch activation negatively regulates proneural wave progression (Figure 3b) [96–103]. In *Su(H)* or *Delta* mutant clones, the proneural wave progression is accelerated [98]. The second Notch activation in NBs regulates the expression of temporal transcription factors in NBs, which defines the neuronal subtype specification [104]. EGFR signalling is activated in the wavefront and induces NB differentiation (Figure 3b, c) [98]. Since Rhomboid (Rho), which is required for EGF ligand secretion, is one of the targets of EGFR signalling, it is speculated that sequential activation of EGFR signalling, which sweeps in a medial to lateral direction, is the driving factor for the progression of the proneural wave [98]. JAK/STAT signalling is activated at lateral NEs and prevents precocious proneural wave progression (Figure 3c) [95,101,105]. Hippo signalling restricts NE proliferation and regulates the NE-NB transition (Figure 3c) [99,106,107].

Although the key components of Notch signalling, such as Delta, Notch, and AS-C are conserved, there are some differences between NB formation in the medulla and that in the embryonic neuroectoderm. First, Delta or AS-C expression does not show any salt-and-pepper pattern during proneural wave progression. Rather, cells at the wavefront show high Notch activity and express L’sc (Figure 3c, f). This is contradictory to the conserved idea of the lateral inhibition mechanism of Notch signalling, which shows a complementary pattern between cells expressing AS-C and cells with high Notch activity. Second, Notch signalling regulates the speed of the proneural wave, and all NEs finally differentiate into NBs following the proneural wave in the optic lobe. This is different from the Notch function in embryonic NB formation, where it regulates binary cell fate choice between neighbouring cells and only a small number of cells differentiate into NBs (Figure 1b).

To elucidate the Notch dynamics in proneural wave progression and interactions among signalling pathways, we and others have developed mathematical models [37,104,108–111]. First, we focused on the interaction between Notch and EGFR signalling pathways because these pathways show transient activation in the transition zone from NEs to NBs and play central roles in proneural wave progression (Figure 3d, e) [98,111]. In the model shown in Figure 3e, *e* represents the concentration of the EGF ligand and activation of EGFR signalling. The secretion of the EGF ligand is spatially restricted by the function of Rho [111]. Since it is feasible to presume the effect of EGFR signalling as a reaction-diffusion system, *E* is positively regulated by EGF ligand diffusion (d_e_ΔE)

nd negatively regulated by degradation (*k_e_E*) [112]. Additionally, *in vivo* experiments showed that activation of EGFR signalling is influenced by AS-C (*a_e_A(A_0_-A)*) (Figure 3d) [111]. *N_i,j_* and *D_i,j_* show the activity of Notch signalling and the amount of Delta in the *i*-th and *j*-th cell, respectively. Since the major components of Notch signalling including Delta, Notch, and AS-C are all involved in proneural wave progression, the effect of Notch signalling is introduced by using a lateral inhibition system into the model. *N_i,j_* is influenced by its degradation (*k_n_N_i,j_), trans*-activation by Delta expressed in neighbouring *l*-th and *m*-th cells (*d_t_Δ_l,m_*_∈Λ*i,j*_*D_l,m_*), and *cis*-inhibition by Delta expressed in the same *i*-th and *j*-th cell (*d_c_D_i,j_*). *D_i,j_* is regulated by its degradation (*k_d_D_i,j_*) and receives a positive input from AS-C (*a_d_A_i,j_*(*A_0_-A_i,j_*)). *A_i,j_* reflects the expression of AS-C including Sc, L’sc, and Ase in the *i*-th and *j-*th cell because these proneural factors show redundant functions in NB differentiation [95]. *A_i,j_* is used as a variable that represents the level of differentiation. *A_i,j_* = 0 in undifferentiated cells, while *A_i,j_* = *A*_0_ in fully differentiated NBs. *A_i,j_* is positively regulated by EGFR signalling (*E_i,j_*), and negatively regulated by Notch signalling (*N_i,j_*) (Figure 3d, e).

Numerical simulations of the model recapitulated the proneural wave progression [111]. Additionally, numerical simulations reproduced loss-of-function phenotypes of EGFR, Notch, or Delta [111]. In the EGFR mutant area, wave progression and NB differentiation did not occur. In contrast, the proneural wave progression was accelerated in the Notch or Delta mutant cells. Therefore, *in silico* analysis combining Notch-mediated lateral inhibition with EGFR-mediated reaction-diffusion explains the *in vivo* proneural wave progression.

Moreover, the mathematical model explained why Notch signalling does not show a salt-and-pepper pattern in proneural wave progression. The absence of the salt-and-pepper pattern in the optic lobe can be explained by the diffusion of EGF ligands. If a cell at the wavefront starts expressing AS-C, it leads to the secretion of EGF ligands. Next, diffusible EGF ligands activate EGFR signalling and induce *AS-C* in neighbouring cells. This function of EGFR signalling may counteract Notch-mediated lateral inhibition and obscure the salt-and-pepper pattern. To test this hypothesis, a computer simulation was performed. In the control condition, *A* and *N* were both activated in the same wavefront cells (figure 3f). However, reduction in EGFR activation showed a complementary pattern of *A* and *N* (figure 3f). This prediction from the simulation was confirmed by the partial knockdown of EGFR activation *in vivo*. Partial disturbance of EGFR function showed a complementary pattern of L’sc and Notch activation. These results suggest that Notch-mediated lateral inhibition is implemented in proneural wave progression and that EGFR-mediated reaction-diffusion cancels salt-and-pepper pattern formation [111].

One may think that Notch activation in the proneural wave progression has a similarity to that in the boundary formation in the *Drosophila* wing margins and veins. In the wing disc, Notch signalling shows a well-aligned activation pattern that defines sharp and stable boundaries through a process distinct from lateral inhibition [32,113–117]. In contrast, Notch-mediated lateral inhibition mediates desynchronization of NB differentiation between neighbouring cells in the optic lobe. Diffusible action of the EGF ligand cancels the salt-and-pepper pattern and forms the boundary of the proneural wavefront that sweeps across the neuroepithelium. Thus, the roles of Notch signalling in boundary formation are different between the wing and the proneural wave.

In the mathematical model described above, Notch signalling shows a single peak at wavefront [111]. Recently, we reported that a modified mathematical model, formulated by introducing strong non-linearity in *cis*-inhibition of Notch signalling, reproduced two peaks of Notch activation *in silico* [104]. *in vivo* experiments showed that Delta expression induces rapid degradation of the Notch protein in late endosomes, which generates a gap between the two Notch activation peaks. Additionally, we showed that the onset of the second Notch activation in NBs regulates neuronal cell fate decisions. These findings demonstrate the molecular mechanism of *cis*-inhibition of Notch signalling and the biological importance of the complex activation mechanism of Notch signalling in neurogenesis [104].

In addition to Notch and EGFR signalling, JAK/STAT and Hippo signalling regulate the proneural wave progression [95,99,101,105–107]. It has been shown that JAK/STAT signalling induces the expression of the Notch target genes in the optic lobe (Figure 3d) [118]. Simulations from a revised mathematical model of the proneural wave including the interaction between Notch and JAK/STAT predicted that JAK/STAT signalling has a noise-cancelling function to suppress random and spontaneous NB differentiation [110]. This prediction from the mathematical model was confirmed by reducing the JAK/STAT activity *in vivo* [110]. Hippo signalling acts upstream of EGFR signalling and mediates NB differentiation by regulating Delta expression (Figure 3d) [99,106]. Future mathematical studies including the function of Hippo signalling will give us a better understanding of the interaction among these signalling pathways and the progression of the proneural wave. Taken together, the combination of mathematical models with *in vivo* experiments using the optic lobe as a model has revealed new functions of Notch-mediated lateral inhibition.

## Conclusion

In this article, we summarized mathematical approaches to elucidate the dynamics of Notch signalling during *Drosophila* neural development. The major roles of mathematical modelling in biology are to reproduce *in vivo* situations and validate the functions of molecules or signalling pathways. In all the cases described above, mathematical and computational simulations accurately recapitulated the *in vivo* patterning. Another important aspect of mathematical modelling is the prediction of new pathway functions and final differentiation patterns by changing parameters or adding new assumptions. *Drosophila* genetics using temperature-sensitive alleles or RNAi lines to partially inactivate gene functions in a spatially and/or temporally restricted manner has enabled the confirmation of mathematical predictions *in vivo* [85,111]. Improving genetic tools to gradually change the activity of target genes and advancing live imaging techniques to acquire quantitative data will be important for validating predictions from mathematical models.

The lateral inhibition mechanism of Notch signalling can be explained by *trans*-activation and *cis*-inhibition. However, *trans*-inhibition and *cis*-activation of Notch signalling have recently been reported. In mammalian angiogenesis, Delta-like 4 activates Notch signalling in neighbouring cells, while another Notch ligand, Jagged1, plays an opposing role [119]. Single-cell imaging in isolated culture cells shows that *cis*-binding of the ligand and receptor activates Notch signalling in a cell [120]. These *trans*-inhibition and *cis*-activation contradict the conserved idea of the lateral inhibition mechanism in which the signal-sending cells activate Notch signalling in neighbouring cells while Notch signalling is inactivated in the signal-sending cells. These mechanisms may be important for fine-tuning Notch activation. Further studies examining the kinetics of ligand-receptor binding and the molecular mechanisms that distinguish whether the binding activates or inhibits Notch signalling are required. Mathematical approaches will also facilitate our understanding of the complicated *trans*- and *cis*- regulations [120–122].

Notch signalling generates different spatial patterns among NBs in the embryo, R8s in the eye disc, and NBs in the optic lobe in *Drosophila*. Another typical pattern generated by Notch signalling is an oscillatory pattern. In vertebrate somitogenesis, an interplay among Notch, fibroblast growth factor (FGF), and Wnt controls the rhythmic production of somites [123,124]. Interdisciplinary research combining *in vivo* experiments with mathematical modelling has revealed the molecular mechanism of how Notch signalling synchronizes oscillatory pattern formation [125–127]. In *Drosophila* eye disc development, decreasing the Notch activity showed a striped differentiation pattern [85]. The numerical simulation of the mathematical model of the proneural wave also showed a striped pattern of NBs when the EGFR activity was decreased, although this has not been reproduced *in vivo* [111]. It is tempting to speculate that the basic function of Notch signalling is mechanistically well conserved in many biological systems, and only a slight difference in Notch activity or interactions between other signalling pathways can generate diverse outputs. Future interdisciplinary research will elucidate the convergence and divergence of Notch dynamics.

## Data Availability

No datasets were generated or analyzed during the current study.
